# Learning curve in full-endoscopic lumbar surgery for disc herniation in a spine clinic in Mexico: Comparative analysis of IELD and TELD techniques

**DOI:** 10.1016/j.bas.2026.106132

**Published:** 2026-06-26

**Authors:** Jose-Carlos Sauri-Barraza, Luis Enrique Nuñez-Alvarado, Eduardo Callejas-Ponce, Jorge Daniel Perez-Ruiz, Eugenio Carral-Robles-Leon, Carlo Enrico Bañuelos-Aluzzi, Luis Manuel Solis-Reyna, Jesus Ernesto Valdez-Aguilar, Francisco Garcia-Muñoz

**Affiliations:** a Centro Médico ABC, Mexico City, Mexico; b Clínica Anglo Americana, Lima, Peru; c Universidad Autónoma del Estado de Hidalgo, Hidalgo, Mexico

**Keywords:** Learning curve, Full-endoscopic surgery, IELD, TELD, CUSUM, Lumbar disc herniation

## Abstract

**Introduction:**

Lumbar disc herniation (LDH) is a common condition that can be treated with full-endoscopic surgery (FES). Publications suggest that the learning curve is steep. There are no reports from Latin America.

**Objective:**

To evaluate the learning curve for FES in LDH and compare outcomes between full-endoscopic interlaminar approach (IELD) and full-endoscopic transforaminal approach (TELD) in Mexico.

**Methods:**

A retrospective cohort of 114 consecutive patients (68 IELD, 46 TELD) was analyzed. Learning curves were modeled using segmented linear regression and Cumulative Sum (CUSUM) analysis to identify transition points in operative time. Clinical outcomes were assessed using the Visual Analog Scale (VAS) for leg/back pain and the Oswestry Disability Index (ODI) at 12 months follow-up. Ridge-penalized logistic regression was used to identify predictors of prolonged surgery.

**Results:**

Technique-specific breakpoints were observed at case 18 for IELD and case 20 for TELD, while the overall learning curve was at 44 cases. The median operative time significantly decreased from the early to late phase (IELD: 120 to 60 min; TELD: 90 to 60 min). Clinical success (≥30% improvement) was achieved by over 76% of patients across all metrics. There were no significant differences in complication and conversion rates between the learning phases.

**Discussion & conclusions:**

Proficiency in FES for LDH was reached after approximately 20 cases per technique. Surgeon experience may influence this learning curve. Both methods yield excellent clinical outcomes. FES is effective, safe, and reproducible in Latin America.

## Highlights

• Regional Benchmark: This study provides the first comprehensive analysis of learning curves for IELD and TELD in a Latin American setting.
• Rapid Proficiency: Segmented regression and CUSUM analysis identified surgical stabilization at 18 cases for IELD and 20 cases for TELD.
• Skill Independence: Mastering one endoscopic approach does not grant immediate proficiency in the other, confirming that IELD and TELD require independent training.
• Safety Thresholds: Reaching the learning breakpoint resulted in a threefold reduction in prolonged operative times and perioperative complications.
• Consistent Outcomes: Clinical improvements in VAS and ODI remained excellent and comparable across both techniques throughout the learning process.
• Expert Influence: The study demonstrates that an experienced microdiscectomy surgeon can achieve a shorter endoscopic learning curve compared to historical novice series.

## 1 Introduction

Lumbar disc herniation (LDH) associated with radiculopathy is a common condition (Sun et al., 2024), with a worldwide prevalence of 1-3% among adults (Hoy et al., 2014). About 15 to 20% of these patients need surgery when conservative treatments do not work (Sun et al., 2024; Weinstein et al., 2006; Katz et al., 2022).

Open microdiscectomy has been the surgical gold standard for treating LDH associated with radiculopathy, with good results in symptom and functional improvement (Weinstein et al., 2008). New minimally invasive techniques, such as lumbar endoscopic surgery, have been shown to offer advantages over microdiscectomy, including shorter hospitalization, faster return to regular activities, less soft-tissue damage, and less need for painkillers, while demonstrating the same clinical benefit (Giordan et al., 2023; Gibson and Waddell, 2007). Expertise in these techniques varies among surgeons based on their training and experience (Olinger et al., 2023; Page et al., 2022).

There are mainly two full-endoscopic lumbar approaches for LDH discectomy: the full-endoscopic interlaminar approach (IELD) and the full-endoscopic transforaminal approach (TELD). The indications and techniques of these approaches have already been well described (Ruetten et al., 2005, 2006; Siepe and Sauer, 2018).

However, the literature reports that IELD and TELD have a longer learning curve, during which complications are more frequent and associated with prolonged surgical times (Ramsay et al., 2001; Wang et al., 2011). According to Koh et al., the average number of cases required to achieve proficiency was 64 for IELD and 59 for TELD (Koh et al., 2025). Articles from Asia, Europe, and the United States (Wang et al., 2011; Koh et al., 2025; Chen et al., 2011; Yeung and Tsou, 2002; Casal-Moro et al., 2010; Ruetten et al., 2008) support this hypothesis, whereas limited studies from Latin America are available.

The objective of this study is to evaluate the learning curve for full-endoscopic lumbar surgery for LDH and to compare learning curve results between the IELD and TELD techniques at a Spine Clinic in a Latin American country.

## 2 Materials and methods

### 2.1 Study design and setting

We conducted a retrospective observational cohort study of consecutive patients undergoing full-endoscopic lumbar spine surgery (IELD or TELD) at ABC Medical Center in Mexico City between January 2017 and June 2023. The study protocol was approved by the Institutional Review Board and the Ethics and Research Committee of ABC Medical Center. All patients provided informed consent for the use of their anonymized data for academic and research purposes.

### 2.2 Population and eligibility criteria

Of 163 surgical cases initially identified, 114 met criteria for analysis. Inclusion: (i) single-level symptomatic lumbar disc herniation (LDH) with clinical–radiologic concordance; (ii) primary full-endoscopic discectomy (IELD or TELD); and (iii) availability of prespecified pre-/postoperative assessments. Exclusion: lumbar spinal stenosis, revision surgery, multilevel disease or combined procedures, and incomplete perioperative data.

### 2.3 Surgical team

All surgeries were performed by a single lead surgeon (J.C.S.B.), a fellowship-trained spine surgeon with 15 years of experience and established proficiency in open and microscopic lumbar surgery. This cohort represents the surgeon's initial experience with full-endoscopic lumbar techniques, ensuring that the learning curve analysis reflects the acquisition of full-endoscopic skills rather than general spine surgical competence. The surgeon was assisted by the same operative team throughout the study period.

### 2.4 Surgical techniques

IELD was performed through the interlaminar window and typically used for central/paracentral herniations; TELD accessed the disc via Kambin's triangle, preferred for foraminal/extraforaminal herniations. All operations used continuous saline irrigation. Given the irrigated endoscopic field, intraoperative blood loss was not quantified. No foraminoplasty or laminectomy was performed. Both approaches followed the surgical techniques described by Ruetten et al., 2005, 2006

### 2.5 Variables and outcome measures

The primary outcome was operative time, measured from skin incision to wound closure. Secondary outcomes were conversion to open microsurgery and intraoperative as well as early postoperative complications within 30 days. Clinical outcomes included back and leg pain on a 0–10 visual analog scale (VAS) and the Oswestry Disability Index (ODI) at preoperative and 12-month follow-up.

### 2.6 Learning-curve analysis

The learning curve was analyzed by technique (within-technique), with separate case sequences for IELD and TELD, and with the overall learning curve (sequence of all cases) for sensitivity analyses. The independent analysis by technique was used to determine whether the learning curves differed across techniques. Learning curves for TELD and IELD were assessed using two complementary mathematical approaches to ensure the robustness of the identified transition points:

#### 2.6.1 Segmented linear regression

Operative time was modeled as a function of chronological case order using a piecewise linear model with a single unknown breakpoint. This breakpoint was identified using a grid search algorithm designed to minimize statistical errors. Confidence intervals (95% CI) for the breakpoint were estimated using nonparametric bootstrap resampling (1000 iterations).

#### 2.6.2 Cumulative sum (CUSUM) analysis

To provide a process-control perspective, we calculated the cumulative sum of deviations from the technique-specific mean operative time. The point of maximal cumulative deviation was defined as the transition from the “learning phase” to “performance stabilization".

##### 2.6.2.1 Sensitivity analysis

To verify the stability of this change-point, CUSUM was repeated using a target (T_0_) defined as the median operative time of the final 20% of cases.

### 2.7 Definition of prolonged surgery

To analyze factors beyond the learning curve, “prolonged surgery” was defined as operative time exceeding the 75th percentile (Q3) for the “late phase” (post-stabilization) of each technique. This threshold ensures that the definition of a “slow” procedure is benchmarked against a surgeon's stabilized proficiency rather than their initial learning period.

### 2.8 Predictive modeling and multivariable analysis

To identify factors associated with prolonged surgery, we employed ridge-penalized logistic regression (L2 regularization). This method was selected to address potential multicollinearity (where variables may be highly correlated, making it difficult to isolate their individual effects on the dependent variable) and to prevent overfitting.

#### 2.8.1 Covariates

The model adjusted for surgical approach (TELD vs. IELD), vertebral level, age, body mass index (BMI), and global case order.

#### 2.8.2 Validation

Model performance was evaluated through two frameworks:

Stratified 5-fold cross-validation
Temporal (forward-chaining) validation: Training on earlier cases and testing on subsequent chronological blocks to account for the inherent “learning effect” over time.

#### 2.8.3 Metrics

Discrimination was quantified using the Area Under the Receiver Operating Characteristic curve (AUC-ROC). Calibration was assessed via decile-based plots and the Brier score.

### 2.9 Subgroup and descriptive statistics

To evaluate whether surgical expertise is solely acquired or if broader knowledge, such as selecting the appropriate surgery for the patient, index level, and type of disc herniation, is also necessary, a pre-specified subgroup analysis was performed for typical indications: IELD for L5–S1 DHs and TELD for L4–L5 DHs. In these groups, operative times were compared between the early phase (before the breaking point) and the late phase (after the breaking point) using medians, interquartile ranges (IQRs), and planning quantiles (P_50_, P_75_, P_90_). Continuous variables are reported as mean (±SD) or median (IQR) based on normality (Shapiro-Wilk test). Categorical data are presented as frequencies and percentages. All tests were two-sided, with statistical significance set at p < 0.05.

### 2.10 Software

Analyses were conducted in Python 3.11 using NumPy, pandas, and custom code for segmented regression, bootstrap, and CUSUM. Figures were generated with matplotlib (one panel per figure; 300 dpi PNG/TIFF), with English axis labels and units.

## 3 Results

### 3.1 Study population

We analyzed 114 consecutive full-endoscopic procedures for lumbar disc herniation: IELD n = 68 and TELD n = 46. TELD patients were older than IELD patients (mean 61.1 vs 48.1 years). Sex distribution was similar (male 51.5% IELD; 45.7% TELD). Index levels differed by technique (IELD: L5–S1 60% [41/68]; TELD: L4–L5 50% [23/46]). Baseline characteristics are summarized in Table 1.

**Table 1:** Baseline characteristics by technique.

Technique	N	Age,mean ± SD (y)	BMI,mean ± SD (kg/m^2^)	Male, n (%)	L5–S1, n (%)	L4–L5, n (%)	L3–L4, n (%)	L2–L3, n (%)
IELD	68	48.1 ± 13.4	24.4 ± 3.2	35 (51.5%)	41(60.3%)	24(35.3%)	2(2.9%)	1(1.5%)
TELD	46	61.1 ± 14.8	25.3 ± 5.6	21 (45.7%)	10(21.7%)	23(50.0%)	8(17.4%)	5(10.9%)

^1^ Values are mean ± SD or n/N (%). IELD = interlaminar endoscopic lumbar discectomy; TELD = transforaminal endoscopic lumbar discectomy.

### 3.2 Learning curve—segmented regression

**Fig. 1:**
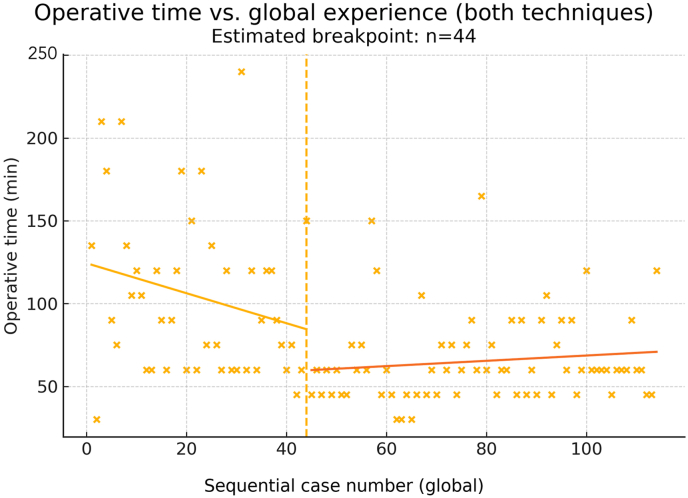
Segmented regression of operative time vs. global case order (both techniques). Breakpoint n = 44 (95% CI 17–79).

**Fig. 2:**
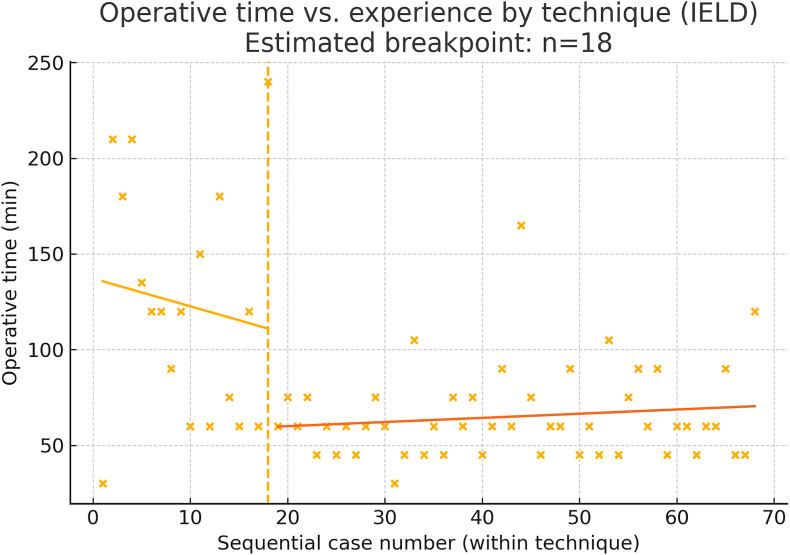

**Fig. 3:**
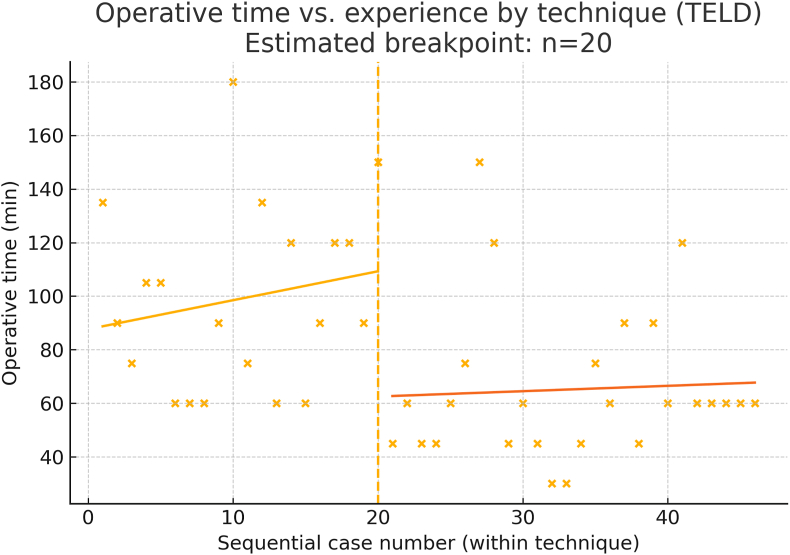

The global (across all cases with both techniques) breaking point occurred at case 44 (95% CI 17–79), with a late-phase (cases after the breaking point) median of 60 min (IQR 45–75; Fig. 1). Technique-specific breakpoints occurred at case 18 for IELD (95% CI 10–39) and case 20 for TELD (95% CI 7–36). Median operative time decreased from 120 to 60 min in IELD and from 90 to 60 min in TELD; late-phase IQRs were 45–75 and 45–71.25 min, respectively (Table 2). Representative fits appear in Fig. 2 (IELD) and Fig. 3 (TELD).

**Table 2:** Technique-specific breakpoints and late-phase operative times.

Technique	N cases	Breakpoint, n (95% CI)	Late-phase median (IQR), min	95th percentile, min
IELD	68	18 (10–39)	60 (45–75.00)	105
TELD	46	20 (7–36)	60 (45–71.25)	120

^1^ Breakpoints from one-knot segmented regression (grid search; ≥15% observations per segment); 95% CIs by bootstrap (300 resamples). Late-phase times as median (IQR) and 95th percentile.

### 3.3 Learning curve—CUSUM

**Fig. 4:**
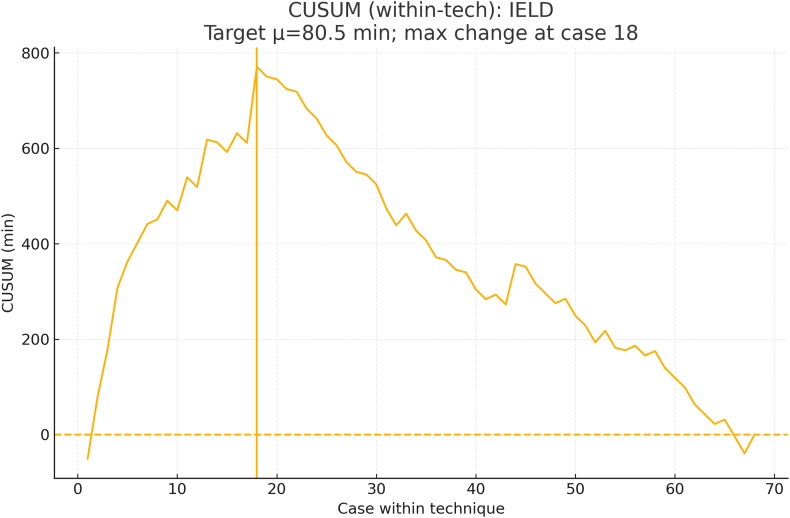

**Fig. 5:**
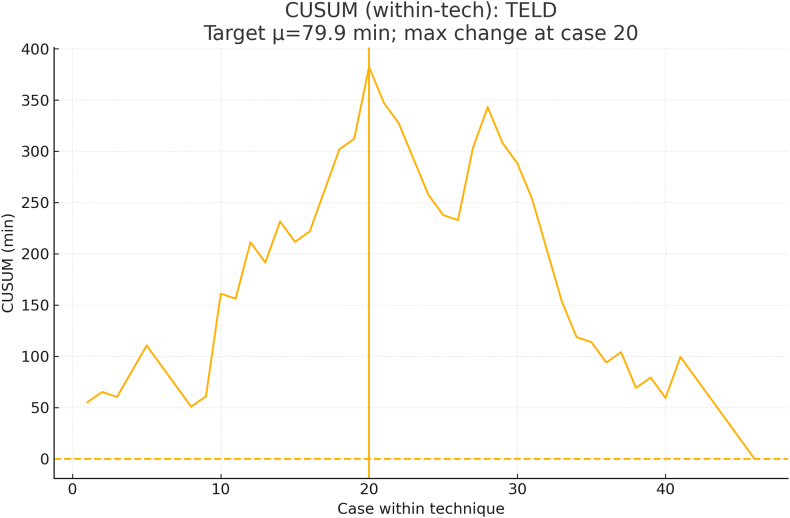

Within-technique CUSUM (mean-target) identified maxima at case 18 for IELD (μ = 80.5 min) and case 20 for TELD (μ = 79.9 min), corroborating the results of the segmented-regression breakpoints (Fig. 4, Fig. 5). A sensitivity CUSUM using the median of the final 20% of cases as target (T_0_ = 60 min) yielded the same change-point locations.

### 3.4 Prolonged procedures (technique-specific thresholds)

Using late-phase Q3 as the technique-specific threshold (surgeries longer than 75 min for IELD and 71.25 min for TLED), the total proportion of prolonged procedures was 30.9% (21/68) for IELD and 47.8% (22/46) for TELD (Table 3). In the early phase (before the

**Table 3:** Prolonged surgery by technique, phase, and level.

Section	Category	N	Prolonged, n (%)
Technique	IELD	68	21 (30.9%)
	TELD	46	22 (47.8%)
Phase within technique	IELD Early	18	12 (66.7%)
	IELD Late	50	9 (18.0%)
	TELD Early	20	15 (75.0%)
	TELD Late	26	7 (26.9%)
Level	L2–L3	6	2 (33.3%)
	L3–L4	10	5 (50.0%)
	L4–L5	47	22 (46.8%)
	L5–S1	51	14 (27.5%)

^1^ Prolonged = operative time > Q3 of the late phase for each technique (IELD = 75.0 min; TELD = 71.25 min).

^2^ 95% CIs are Wilson intervals (reported only for 'Phase within technique').

^3^ Late phase is defined as cases after the segmented-regression breakpoint.

breaking point), prolonged procedures were more common than in the late phase (after the breaking point): IELD 66.7% vs 18.0%; TELD 75.0% vs 26.9%, respectively. By level, prolonged procedures are described in Table 3.

### 3.5 Discrimination of other factors that may influence prolonged surgeries

In the primary ridge-penalized logistic model, experience was independently protective (OR 0.97 per case, 95% CI 0.96–0.99, p < 0.001). Comparing L4–L5 vs L5–S1, L5-S1 showed a trend toward higher odds of prolonged procedures (OR 2.41, 95% CI 0.90–6.42, p = 0.079); estimates for L3–L4 and L2–L3 were imprecise. The technique chosen (TELD vs IELD), global case order, age, and BMI were not associated with prolonged surgeries. The sensitivity model, including an explicit early/late indicator and a binary L5–S1 term, compares L5-S1 against the combined other levels to create two larger groups for comparison, thereby strengthening the statistical analysis. The early phase strongly predicted prolonged surgery (OR 25.31, 95% CI 4.03–159.1, p < 0.001). Full results are reported in Table 4, Table 5.

**Table 4:** Primary model: Adjusted associations with prolonged operative time.

Variable	OR (95% CI)	p value
Surgical approach		
TELD vs IELD	0.83 (0.30–2.34)	0.729
Vertebral level (reference: L5–S1)		
L4–L5	2.41 (0.90–6.42)	0.079
L3–L4	4.65 (0.83–26.15)	0.081
L2–L3	2.18 (0.24–19.63)	0.487
Experience and patient factors		
Cumulative experience (per case)	0.97 (0.96–0.99)	<0.001
Age (per year)	1.02 (0.99–1.06)	0.140
BMI (per kg/m^2^)	1.05 (0.95–1.16)	0.325

∗Prolonged operative time was defined as duration above the technique-specific 75th percentile of the late learning phase. Vertebral level was modeled using categorical indicators with L5–S1 as reference. Odds ratios were estimated using ridge-penalized logistic regression (L2).

**Table 5:** Sensitivity model: Learning-phase–adjusted associations with prolonged operative time.

Variable	OR (95% CI)	p value
Surgical approach		
TELD vs IELD	0.72 (0.24–2.17)	0.563
Patient factors		
Age (per year)	1.03 (1.00–1.07)	0.074
BMI (per kg/m^2^)	1.06 (0.96–1.17)	0.255
Learning-related variables		
L5–S1 (protective level)	0.46 (0.16–1.32)	0.149
Early learning phase (vs late)	25.31 (4.03–159.1)	<0.001

∗The sensitivity model replaces vertebral-level indicators with a binary L5–S1 variable and includes early/late learning phase. Cumulative experience (case order) was excluded to avoid collinearity and outcome-definition leakage. Odds ratios were estimated using ridge-penalized logistic regression (L2).

### 3.6 Discrimination and calibration of the prolonged-duration classifier

#### 3.6.1 Continuous-time prediction

When operative time was modeled as a continuous variable, out-of-sample performance was limited. A linear model that includes experience, technique, level, age, and BMI achieved an R^2^ ≈ 0.11 and an MAE ≈22–23 min under stratified 5-fold CV; forward chaining yielded an R^2^ ≈ 0.10, suggesting that the results at the breaking point are reliable and not influenced by other factors. A log-time specification with a technique L5–S1 interaction increased R^2^ to ≈0.18 and reduced MAE by ∼2 min.

#### 3.6.2 Classification of prolonged procedures

**Fig. 6:**
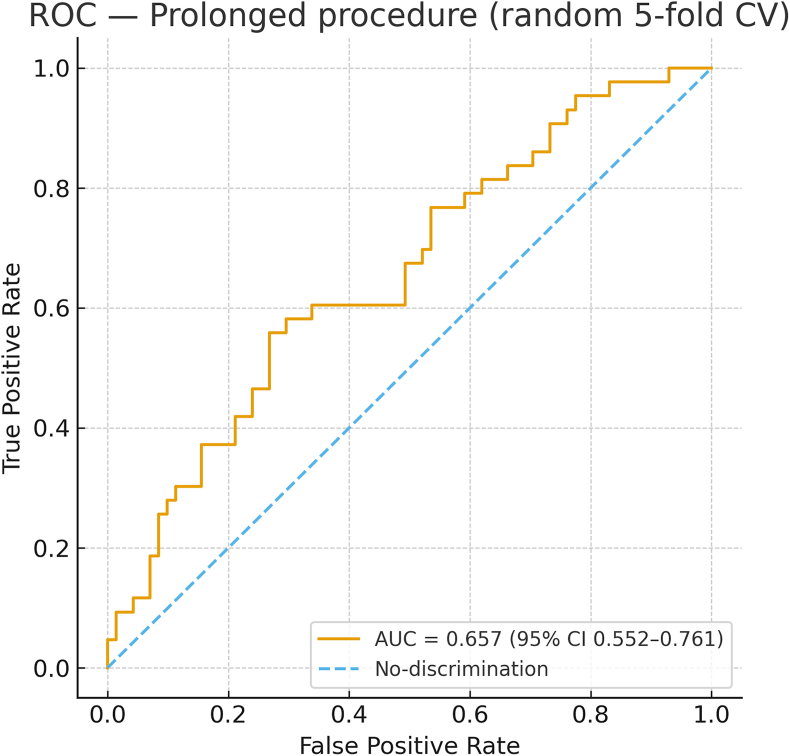

**Fig. 7:**
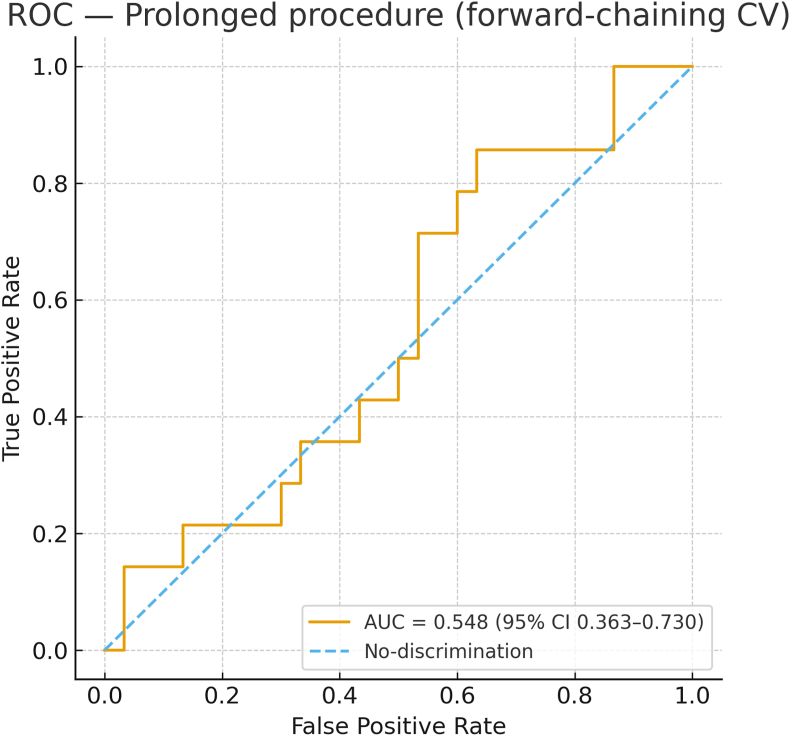

Reframing the endpoint as “prolonged” (time > late-phase Q3 within technique) produced modest out-of-sample discrimination. In stratified 5-fold CV, AUC = 0.67 (95% CI 0.55–0.77) with Brier = 0.218; in forward-chaining, AUC = 0.58 (95% CI 0.45–0.71) with Brier = 0.229 (Table 6; Fig. 6, Fig. 7). The lower AUC under temporal validation is expected in learning-curve settings and reflects temporal non-stationarity.

**Table 6:** Cross-validated discrimination and calibration for prolonged-duration classification.

Validation scheme	AUC (95% CI)	Brier score	Held-out N
Random 5-fold CV (stratified)	0.67 (0.57–0.77)	0.218	114
Forward-chaining CV (temporal)	0.58 (0.45–0.71)	0.229	91

^1^ ROC/AUC computed from pooled out-of-sample predictions. 95% CIs by nonparametric bootstrap on held-out predictions. Brier score summarizes calibration error (lower is better).

### 3.7 Conversions and complications

Conversions to microdiscectomy occurred in 6 of 68 IELD cases (8.8%), with none in TELD. In IELD, all conversions occurred pre-breakpoint in the early phase (6/18 = 33.3%; 95% CI 16.3–56.3), whereas none occurred after the breakpoint in the late phase (95% CI upper bound 7.1%). Overall, the complication rate was 3.5% (4/114). For IELD, there was 1 complication out of 18 cases in the early phase (5.6%) and 1 out of 50 cases in the late phase (2.0%). For TELD, 1 complication was observed in 20 cases in

the early phase (5.0%) and 1 in 26 cases in the late phase (3.85%) (Table 7). Given the low number of complications, multivariable models are recommended, and proportions are reported with Wilson 95% CIs.

**Table 7:** Conversions and complications by technique and phase.

Technique	Phase	N	Conversi ons, n (%)	Conv. 95% CI (%)	Complications, n (%)	Comp. 95% CI (%)
IELD	Early	18	6 (33.3%)	16.3–56.3	1 (5.6%)	1.0–25.8
IELD	Late	50	0 (0.0%)	0.0–7.1	1 (2.0%)	0.4–10.5
TELD	Early	20	0 (0.0%)	0.0–16.1	1 (5.0%)	0.9–23.6
TELD	Late	26	0 (0.0%)	0.0–12.9	1 (3.8%)	0.7–18.9

^1^ Proportions with Wilson 95% CIs. Low event counts precluded multivariable models for complications.

### 3.8 Clinical outcomes

At 12 months, both techniques demonstrated clinically meaningful improvement, with no statistically significant difference, as shown in Table 8.

**Table 8:** Clinical outcomes at 12 months (VAS-leg/back, ODI).

Technique	VAS-leg Δ12m, mean ± SD [median, IQR]	≥30% improvement (VAS-leg), %	VAS-back Δ12m, mean ± SD [median, IQR]	≥30% improvement (VAS-back), %	ODI Δ12m (0–1), mean ± SD [median, IQR]	≥30% improvement (ODI), %
IELD	−4.40 ± 3.08 [-5, -7–-3]	76.5%	−5.41 ± 2.36 [-6, -7–-4]	92.6%	−0.184 ± 0.159 [-0.18, −0.29–-0.08]	79.4%
TELD	−4.65 ± 2.99 [-5, -7–-3]	76.1%	−5.63 ± 1.90 [-6, -7–-5]	95.7%	−0.184 ± 0.136 [-0.17, −0.26–-0.10]	87.0%

^1^ Changes reported as mean ± SD [median, IQR] from baseline; ≥30% improvement proportions shown. ODI recorded as proportion (0–1).

## 4 Discussion

The breaking point on the learning curve in this study occurred at case 18 for IELD and at case 20 for TLED, which is earlier than in some other papers in the literature (Koh et al., 2025; Jitpakdee et al., 2023; Jamaleddine et al., 2025; Wang et al., 2024; Son et al., 2020; Fleiderman et al., 2023). To confirm these results, a robust statistical analysis was performed. We used segmented linear regression analysis, a common method in such studies. However, on its own, it is not highly reliable; therefore, a CUSUM analysis was added to strengthen confidence in the results (Lin et al., 2023; Shahi et al., 2023; Maayan et al., 2023; Xu et al., 2022; Wu et al., 2024; McNamee et al., 2024; Papachristofi et al., 2016; Yan et al., 2022). Additionally, to account for factors that may affect prolonged surgery unrelated to the learning curve, a ridge-penalized logistic regression (L2 regularization) was used. To discriminate the data, ROC/AUS methods were developed (Lin et al., 2023; Shahi et al., 2023). Random resampling was excluded from cross-validation, and calibration was evaluated using decile-based calibration plots and the Brier score. Because there is a potential bias or methodological error (outcome-definition circularity) due to the outcome being included in the predictor variables, since the early and late phase classifications were based on operative-time trajectories, a sensitivity analysis was conducted to validate the previous statistical results. All this data confirms that the results are reliable.

Even though there are anatomical and technical differences between the IELD and TELD approaches, the gap between the two braking points is narrow, consistent with findings in the literature (Jitpakdee et al., 2023; Zhou et al., 2021; Zhang et al., 2023).

As shown in other publications (Krzok, 2020; Ali et al., 2023; Álvarez de Mon-Montoliú et al., 2025), the shortest learning curves have been associated with experienced surgeons and high institutional surgical volume, as in this study. The lead surgeon is a fellowship-trained spine surgeon with 15 years of practice, focused on minimally invasive techniques. These results reflect the learning curve of an experienced surgeon performing de novo full-endoscopic surgery, not that of a novice spine surgeon; therefore, caution is warranted when interpreting this paper, and one may expect a longer learning curve in young surgeons. Therefore, accompanied mentoring during the learning curve for inexperienced spine surgeons is highly recommended (Park et al., 2025; Wu et al., 2020).

The global endoscopic learning curve had a breakpoint at 44 cases, reflecting the cumulative volume required to master the full spectrum of lumbar full-endoscopic surgery for LDH. Because patients were added to the study chronologically, both techniques were evolving simultaneously, with a technique-specific learning curve around case 20 and a total of 44 cases across both techniques. This finding highlights that mastering one approach does not immediately confer proficiency in the other (Xu et al., 2022; Álvarez de Mon-Montoliú et al., 2025; Liu et al., 2024), underscoring the importance of a modular curriculum that ensures competency in both anatomical corridors (Schmidt et al., 2020; Falavigna et al., 2020).

Post-braking-point median operative time of 60 min for both approaches aligns with benchmarks from high-volume centers and meta-analyses (Koh et al., 2025; Jamaleddine et al., 2025; Son et al., 2020), and the late-phase 95th percentiles (105 min IELD; 120 min TELD) remain clinically acceptable.

The IELD technique was most commonly used at L5–S1 (60%), where the interlaminar corridor is accessible, while TELD was more frequently used at L4–L5 (50%), where foraminal access is clear of the iliac crest (Jitpakdee et al., 2023; Zhang et al., 2023; He et al., 2021; Hua et al., 2018). These patterns support the idea that approach choice should be based on anatomy rather than age (Jitpakdee et al., 2023; Zhang et al., 2023; He et al., 2021).

Age (which has been linked to increased spinal degeneration), BMI, the involved index level, and other demographic factors did not influence the learning curve, indicating that experience and choosing the right approach are more important, aligning with the literature (Eun and Oh, 2024; Son et al., 2023; Kim et al., 2018). Additionally, this suggests that full-endoscopic surgery may have an advantage over other techniques, where age (greater spinal degeneration) and BMI are negative prognostic factors (Eun and Oh, 2024; Son et al., 2023; Kim et al., 2018; Ferguson et al., 2021; Kobayashi et al., 2019).

A threefold reduction in “prolonged” surgeries and complications was observed after the learning curve breakpoint. These findings are consistent with systematic reviews (Ali et al., 2023; Álvarez de Mon-Montoliú et al., 2025; Park et al., 2025; Wu et al., 2020). No conversions to microsurgery occurred after case 18 in IELD (TLED had none). Our complication rate (3-5%) aligns with the 3–8% rates reported in other historical series, a pattern now lessened by staggered training and mentoring [18,40,41,69,71,72]. Our complication rates are similar to those of microdiscectomy. These data support that once proficiency is achieved, safety and effectiveness remain consistent (Jitpakdee et al., 2023; Park et al., 2025; Wu et al., 2020; Yang et al., 2022; Hsu et al., 2013; Boadi et al., 2024).

These complications and conversions did not negatively impact the patients' outcomes, demonstrating that the surgeon managed the issues effectively. This indicates that experience and familiarity with surgical techniques beyond endoscopy are valuable resources for handling complications (Wu et al., 2022; Chen et al., 2024).

There was no difference in outcome between the two techniques, indicating that the pathology was resolved in both cases. Even in prolonged cases on the learning curve, the results remained positive, suggesting that surgical time is not a prognostic factor for patient outcomes in full-endoscopic spine surgery for LDH.

## 5 Conclusions

Our cohort analysis shows that operative efficiency stabilized after a learning phase, with a global breakpoint at case 44. For IELD, stabilization occurred after case 18, and for TELD, after case 20. This suggests that gaining experience with one technique does not confer expertise in the other, requiring an independent learning curve. Although the learning curve was brief in this study compared with other series worldwide, these results align with other publications reporting outcomes achieved by experienced spine surgical teams and high surgical volumes, as in our study. It is expected that the learning curve will be longer for young spine surgeons or those with low spine surgery volumes; therefore, mentoring with an experienced surgeon is recommended.

When comparing ILED and TLED, clinical outcomes showed significant improvement, with no statistical difference between the two and no difference in the learning curve. However, there was a clear difference in conversion to microsurgery, observed only in the early phase with ILED. The complication rate was similar between techniques and remained low throughout the study. The surgeon's experience may contribute to the low complication rate.

This paper confirms that full-endoscopic spine surgery for LDH is effective, safe, and reproducible in Latin America, with learning curves similar to those in Europe and Asia.

## Declaration of competing interest

The authors declare that they have no known competing financial interests or personal relationships that could have appeared to influence the work reported in this paper.
