# Supplementary material for: Learning curve in full-endoscopic lumbar surgery for disc herniation in a spine clinic in Mexico: Comparative analysis of IELD and TELD techniques
Source: Brain Spine. 2026 Jun 26;6:106132. doi: 10.1016/j.bas.2026.106132 (PMC13329384; doi:10.1016/j.bas.2026.106132)
Supplement: Multimedia component 1 [file mmc1.docx]

**SUPPLEMENTARY APPENDIX**

**Article Title:** LEARNING CURVE IN FULL-ENDOSCOPIC LUMBAR SURGERY: COMPARATIVE ANALYSIS OF IELD AND TELD TECHNIQUES.

**Manuscript Number:** BAS-D-25-00264R1

**S1. MATHEMATICAL SPECIFICATIONS OF LEARNING CURVE ANALYSIS**

**1. Segmented (Piecewise) Linear Regression**

For each technique, operative time *y* was modeled against case order *x* with a single unknown breakpoint *τ*:

*y* =  *β*_0_^(^*^E^*^)^ + *β*_1_^(^*^E^*^)^*x*, *x ≤ τ,*

*β*_0_^(^*^L^*^)^ + *β*_1_^(^*^L^*^)^*x*, *x > τ*

The breakpoint *τ* was selected by grid search minimizing the total sum of squared errors with a constraint of ≥15% of observations per segment. 95% confidence intervals for *τ* were obtained by nonparametric bootstrap (resampling pairs with order restored by sorting on *x*; 300 resamples). For interpretability, we summarized medians and interquartile ranges (IQRs) before and after *τ*.

**S2. CUSUM (CUMULATIVE SUM) ANALYSIS**

The CUSUM method was used as a process-control tool to detect deviations from the process mean, identifying the point where the learning process stabilizes.

Within each technique, cases were ordered chronologically and operative times {t_i_} centered on the technique-specific mean μ. The process metric was:

CUSUM*k* = Σ*i* = 1^k^ (*t*_i_-μ), CUSUM_0_= 0.

The index of maximal CUSUM was used as a data‑driven change‑point (i.e., case with the largest cumulative deviation before the curve turns downward toward stabilization). CUSUM is a process‑control metric of operative time; it does not directly inform on safety or clinical effectiveness. Sensitivity analysis. We repeated CUSUM using a target *T*_0_ defined as the median of the last 20% of cases within each technique to verify the robustness of the change‑point to the choice of target.

We compared the CUSUM change-point and the segmented-regression breakpoint for consistency, acknowledging that both quantify time-based learning rather than complication-related proficiency.

**S3. PENALIZED LOGISTIC REGRESSION (RIDGE)**

To identify factors associated with "prolonged" surgery (operative time > 75th percentile of the late phase) while avoiding overfitting due to the relatively small sample size and potential separation (e.g., zero conversions in late phase), we utilized **Ridge Logistic Regression** (L2 penalization).

Standard logistic regression estimates coefficients β by maximizing the log-likelihood L(β). In Ridge regression, a penalty term proportional to the square of the coefficients is subtracted from the log-likelihood:

**
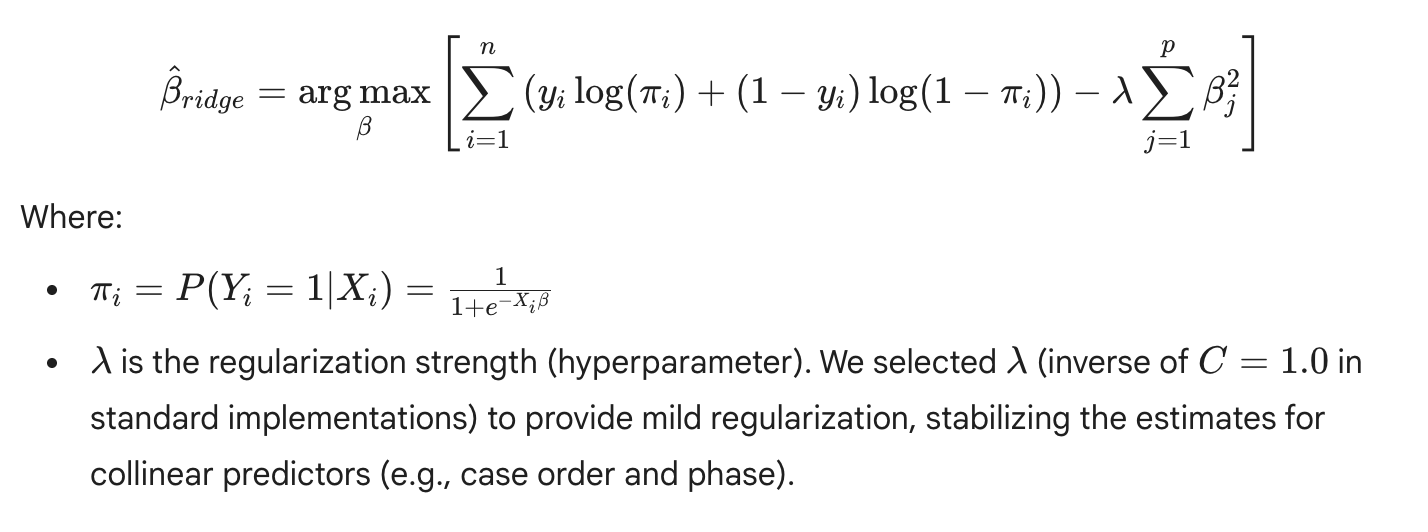
**

Where:

- π_i_ = P(Y_i_=1|X_i_) =1/1 + e^-Xiβ^
- λ is the regularization strength (hyperparameter). We selected λ (inverse of C=1.0 in standard implementations) to provide mild regularization, stabilizing the estimates for collinear predictors (e.g., case order and phase).

**S4. SUPPLEMENTARY TABLES**

**Table S1. Subgroup summaries in typical indications (IELD at L5–S1; TELD at L4–L5)**

| **Technique** | **Level** | **N** | **Early N** | **Early Median (IQR)** | **Late N** | **Late Median (IQR)** | **Prolonged, n (%)** |
| --- | --- | --- | --- | --- | --- | --- | --- |
| **IELD** | L5-S1 | 41 | 11 | 120.0 (60.0 – 120.0) | 30 | 60.0 (45.0 – 60.0) | 8 (19.5%) |
| **TELD** | L4-L5 | 23 | 12 | 90.0 (71.3 – 120.0) | 11 | 60.0 (45.0 – 67.5) | 12 (52.2%) |

Note: Operative times are in minutes. "Prolonged" is defined relative to the technique-specific late-phase Q3 threshold.
